# Development and validation of prechiasmatic mouse model of subarachnoid hemorrhage to measure long-term neurobehavioral impairment

**DOI:** 10.21203/rs.3.rs-4176908/v1

**Published:** 2024-04-02

**Authors:** Deepti Diwan, Jogender Mehla, James W. Nelson, Gregory J. Zipfel

**Affiliations:** Washington University School of Medicine; Washington University School of Medicine; Washington University School of Medicine; Washington University School of Medicine

## Abstract

Controllable and reproducible animal models of aneurysmal subarachnoid hemorrhage (SAH) are crucial for the systematic study of the pathophysiology and treatment of this debilitating condition. Despite the variety of animal models of SAH currently available, attempts to translate promising therapeutic strategies from preclinical studies to humans have largely failed. This failure is likely due, at least in part, to poor replication of pathology and disabilities in these preclinical models, especially the long-term neurocognitive deficits that drive poor quality of life / return to work in SAH survivors. Therefore, there is an unmet need to develop experimental models that reliably replicate the long-term clinical ramifications of SAH – especially in mice where genetic manipulations are straightforward and readily available. To address this need, we developed a standardized mouse model of SAH that reproducibly produced significant and trackable long-term neurobehavioral deficits. SAH was induced by performing double blood injections into the prechiasmatic cistern – a simple modification to the well-characterized single prechiasmatic injection mouse model of SAH. Following SAH, mice recapitulated key characteristics of SAH patients including long-term cognitive impairment as observed by a battery of behavioral testing and delayed pathophysiologic processes assayed by neuroinflammatory markers. We believe that this new SAH mouse model will be an ideal paradigm for investigating the complex pathophysiology of SAH and identifying novel druggable therapeutic targets for treating SAH-associated long-term neurocognitive deficits in patients.

## Introduction

Spontaneous subarachnoid hemorrhage (SAH) from rupture of an intracranial aneurysm leads to significant mortality and long-term neurological deficits, with ~ 50% of SAH patients suffering major cognitive deficits precluding their return to work or resumption of daily activities[1–3]. Several methods for modeling SAH have been developed in an effort to provide an experimental foundation for mechanistic and therapeutic investigation in the preclinical setting. Most of these animal models recapitulate some, but not all, of the acute pathophysiological events that occur after SAH, including development of secondary brain injury from Early Brain Injury (EBI) and Delayed Cerebral Ischemia (DCI)[4]. Less well characterized are the long-term consequences of SAH, especially its deleterious impact on cognitive function.

The most frequently used animal models of SAH are: (i) Endovascular perforation model, which involves advancing a suture into the internal carotid artery (ICA) until it perforates a vessel within the Circle of Willis leading to extravasation of blood into the basal cisterns[5–9]; and (ii) Direct injection of blood into the cisterna magna or prechiasmatic cistern[10–12], where the former results in a blood clot primarily localized around vessels of the posterior circulation and the latter around vessels of the anterior circulation[13, 14]. Each of these models has their own unique set of advantages and disadvantages, but none fully recreate the pathologic conditions of human SAH[15–17]. One of the many reasons for this situation could be that rodent models of SAH are technically demanding and have been difficult to standardize for variability in hemorrhage severity[18].

Regarding long-term cognitive deficits after experimental SAH, a variety of induction methods, rodent species, and timepoints have been examined. To date, the most consistent and trackable neurocognitive deficits have been demonstrated in rat models of SAH including endovascular perforation[19], prechiasmatic injection[20], and cisterna magna injection[20, 21] techniques. All three induction methods produce significant neurocognitive deficits in rats up to 1-month after ictus. However, rats are more expensive than mice and targeted genetic manipulation is significantly more challenging. In contradistinction, long-term neurocognitive deficits in mouse models of SAH have proven signficantly more challenging. For prechiasmatic injection and cisterna magna injections mouse models, neurocognitive deficits have only been demonstrated up to 2-week after ictus (Table 1)[12, 22, 23]. For the endovascular perforation mouse model, disparate results have been reported with Regnier-Golanov *et al*. reporting significant neurocognitive deficits 1-month after ictus using a battery of behavioral tests including Morris Water Maze [24], while Milner *et al*. showed no neurocognitive deficits 1-month after ictus as assessed by Morris Water Maze[25] and Fanizzi et al. showed minimal long-term neurobehavioral deficits 1-month after ictus using a battery of behavioral tests including Morris Water Maze[26]. Development of a mouse model of SAH that produces robust and consistent long-term neurocognitive deficits is highly desirable, as it would accelerate experiments, lower costs, and leverage the tremendous power of genetically manipulated mice.

To address this important investigational gap, we developed a “prechiasmatic double injection” mouse model of SAH in which heterologous arterial blood is injected twice into the prechiasmatic cistern 24h apart. Using this model, we performed a validation study using a battery of well-characterized behavioral tests to define long-term neurocognitive deficits (> 30 days post-SAH) and corroborated these deficits with chronic neuroinflammatory changes as assessed by immunohistochemistry.

## Materials and Methods

### Animals

All experimental procedures were carried out following the guidelines for the care and use of animals in research, which were established by the Institutional Animal Care and Use Committee at Washington University and approved by the National Institutes of Health. Male C57BL/6J mice between 12–14 weeks old were obtained from Jackson Laboratories (Bar Harbor, ME). The mice were provided ad libitum access to water and a standard mouse chow diet, and were acclimated to a controlled environment with a 12-hour light/dark cycle. The animals were randomly assigned to experimental groups.

### SAH induction in mice

Experimental SAH was induced in mice using the prechiasmatic injection technique. Briefly, the mice were first anesthetized with isoflurane (2% induction, 1.5% maintenance) in a mixture of 70:30 N_2_O: O_2_, and their body temperature was maintained at 37°C with a rectal temperature probe and a thermo-regulated heating pad. The mice were then secured in a stereotaxic frame, and the scalp was incised to expose the surface of the dorsal skull. An intracranial pressure (ICP) probe was placed to monitor ICP during and after the procedure. A hole was made in the atlanto-occipital membrane using a 23G needle to insert the ICP probe. The probe was pulled slightly to ensure that it showed a pulsating curve ranging between 0–5 mmHg.

For SAH induction, an incision was made in the midline of the anterior scalp, exposing the transparent skull. The superior sagittal sinus was observed and avoided during SAH induction. A 0.7 mm burr hole was drilled in the skull 5 mm anterior to the bregma and slightly off the midline to avoid the sagittal sinus. At this point, 0.1 ml of fresh heparinized blood was collected from a littermate donor. A 27G spinal needle connected to a 1cc syringe was positioned with the side port facing up and angled 32° from the vertical axis. The needle was advanced carefully until contact was made with bone at a depth of about 7 mm, and then the needle was retracted 0.5–1.0 mm. 100μl of blood for SAH induction or artificial cerebrospinal fluid (CSF) for sham controls was injected through the spinal needle over 40–45s using a digital infusion pump. The needle was kept in place for an additional 2–3 min to prevent backflow of injected blood or CSF leakage, then slowly withdrawn. The second blood injection was performed at the same site 24 h after the first injection using the same protocol. Following surgery, any residual blood products or debris were cleared from the skull surface. The drilling point was sealed with bone wax, and the incision was closed with a sterile suture. The mice were allowed to recover from anesthesia in an incubator and then returned to their cages.

Mice were subjected to transcardial perfusion with heparinized PBS, and the brain was carefully removed to examine the blood distribution along subarachnoid vessels. No visible blood was observed in the sham-operated animals. In contrast, in the case of SAH, extravasated blood was consistently found covering the MCA and distributed along larger arteries that branch from the MCA. Any animals that died during or immediately after the surgical procedure, those from the SAH group without subarachnoid blood, those from the sham group with subarachnoid blood, and those with severe hemiparesis within 6h of surgery (indicative of inadvertent MCA occlusion) were excluded from the analysis. All SAH and sham-operated animals that survived and were able to complete neurobehavioral assessments were included in the analysis.

### Behavioral assement methods

#### Morris Water Maze

The Morris water maze was performed in accordance with our previous studies[27–29]. Briefly, two principal axes of the maze are the standard designation; with each line bisecting the maze is perpendicular to the another, dividing it into four imaginary cardinal poles: North (N) – opposite point of the experimenter, South (S) – experimenter’s position, east (E) – experimenter’s right and West (W) – experimenter’s left; thereby creating four quadrants.

A pool filled with opaque, non-toxic white paint was used for all experiments. An escape platform was placed in one of the quadrants of the pool beneath the water surface (0.5–1 cm), located halfway between the wall and the center of the pool. The water temperature was maintained at 22 ± 1°C to prevent the mouse from floating. Three cues of different shapes were placed around the water pool to help the mouse in finding the platform. Mice were randomly placed in the water, and an automated ANY-Maze Behavioral Tracking Software (Stoelting) was used to record the time taken to reach the escape platform (escape latency), swim speed, and total swim distance. The mice underwent four trials per day at each of the four cardinal drop points (north, south, east, west) in random order for eight consecutive days, with the platform hidden at the same location each day. If the mice failed to locate the escape platform within the 60-second trial length, mice were manually transferred to the platform for around 15 seconds. A single probe trial without a hidden platform was completed on the 9th day. To ensure no visual deficit due to SAH surgery, a visible platform test of the MWM was also performed.

#### Y-maze

The Y-maze test is a method used to evaluate spatial working and reference memory in rodents, as described in previous studies [27–29]. Briefly, The test involves a Y-shaped maze consisting of three white, opaque plastic arms at a 120° angle. The mice were placed in the center of the maze and allowed to explore two arms for 10 min during the training trial. The third arm (novel arm) was kept closed (Fig. 4a,b). After a 30-minute inter-trial interval, the blocked arm was opened, and the mice were placed again in the center of the maze to explore all three arms for 10 min during the test trial. Over the course of multiple arm entries, the animal should display a tendency to enter the third arm that was less visited. The number of arm entries and triads was recorded to calculate the percentage of alternation. An entry is defined as when all four limbs are within the arm, and the total number of novel arm entries by mice is then calculated.

#### Novel Object Recognition (NOR)

The NOR test is a widely used method for assessing the memory of rodents. The test is based on the innate inquisitiveness of rodents to explore novel objects and retain their spatial memory [28–31]. The test was conducted in an open-field arena, to which the mice were first habituated. On the next day, four objects made of similar materials but differing shapes were placed within the arena, spaced equidistantly with an empty space for the mice to explore. The mice were then introduced into the arena with two identical objects and allowed to explore for 10 minutes. The mice were then removed, and one of the two identical objects was replaced with a novel object. Following a 10-minute inter-trial period, the mice were returned to the arena, and their exploration behavior for novel objects was recorded using a video camera for 5 min. The mice were considered to be engaged in object exploration when their head was oriented within 45° of an object and within 4 cm of it. Rearing with the head oriented upward was also included if at least one forepaw was on the object. However, climbing over or sitting on the objects was not included. Finally, the percentage of exploratory preference was calculated.

#### Fear Conditioning (FC)

FC test was conducted to evaluate amygdala- and hippocampus-dependent memory in rodents, as described in our previous publications [27, 29, 31]. The test was carried out in an acrylic square chamber (33 × 33 × 25 cm) with a stainless steel rod floor that was connected to a shock generator for delivering a footshock. A tone stimulus was delivered through a speaker. Before conditioning, the chamber was cleaned using a 1% Virkon solution to mask any former odor cues. On the day of conditioning, mice were transported from their home cage to a testing room and allowed to sit undisturbed in their cages for 10 minutes. Mice were then placed in the conditioning chamber and allowed to explore for 2 minutes before the onset of the tone (20 seconds, 2000 Hz). In the delay conditioning procedure, a 2-second and 0.5 mA shock was given in the last 2 seconds of the tone duration. Mice recieved five delayed conditioning trials, with 120 seconds intertrial intervals (ITI). One minute after the last shock, the mice were removed from the conditioning chamber and returned to their home cages. After 24 hours, the tone test was conducted in a triangular chamber situated in a different room. The chamber was geometrically different from the conditioning chamber, thereby enabling the assessment of the tone conditioning in the absence of the training context. The chamber was cleansed with 70% isopropyl solution after every mouse. During the tone test, three 20-second tones were given after a 2-minute baseline period, with each tone separated by a 120-second ITI. The mice were removed from the triangular chamber 1 minute after the last tone presentation and returned to their home cages. The freezing response was measured using a time sampling procedure, in which an observer scored the presence or absence of the freezing response for each mouse at every 2-second interval. Twenty-four hours after the tone test, a context test was conducted by placing each mouse back in the original conditioning chamber for 5 minutes. During this test, freezing was scored for each mouse at every 5 second interval. The data were transformed into a percentage freezing score by dividing the number of freezing observations by the total number of observations and multiplying by 100.

#### Immunohistochemistry

Immunohistochemistry was performed as previously described [6, 8, 27–29, 32]. Free-floating brain sections with a thickness of 40-μm were subjected to fluorescent immunohistochemical staining using the following primary antibodies: anti-mouse fibrinogen (ab34269, 1:1000 dilution), anti-Iba1 monoclonal antibody (MA5–27726, 1:1000 dilution), anti-P2RY12 monoclonal antibody (MABN2593, 1:1000 dilution), and anti-glial acidic fibrillary protein (GFAP) (IF03L, 1:1000 dilution). The secondary antibodies used included Alexa Fluor 488, 647, and 594 Goat Anti-mouse IgG. In brief, free-floating brain sections were washed with TBS and then blocked in TBS (prepared in 0.3% Triton-X 100 and 3% goat serum) for 2h. Subsequently, the brain sections were incubated in primary antibodies while being shaken at room temperature for 24h. Three 10-minute washes were performed after 24h, and the brain sections were incubated in secondary antibodies for another 24h. After incubation, the brain sections were washed three times for 10 minutes each. Next, brain sections were then mounted on slides using Vectashield H-1000 (Vector Laboratory), and the slides were sealed with nail polish. Finally, the samples were imaged using a Nikon Eclipse ME600 digital video microscopy system (Nikon Instruments Inc., Melville, NY), and the images were analyzed with Image-J software.

#### Statistical analysis

Statistical analysis was performed using GraphPad Prism 9.0 (GraphPad Software Inc., CA, USA). We estimated sample sizes using effect sizes based on our previous publications[27–29, 31] and pilot experiments. The sample size for the two-sample t-test was four to five per group. In addition, we recommend having a backup animal in case of possible accidents during the procedure, for example, skull damage during ear bar mounting, which can cause intracranial bleeding that may affect the final outcome of the experiment. An independent two-tailed t-test was used to obtain statistically significant differences between the groups. All data were tested for normality using the Shapiro-Wilk test. Results represented as mean ± s.d. Statistical significance was set at P < 0.05.

## Results

A standardized rodent model that closely replicates human pathophysiology of SAH including reliable and trackable long-term neurocognitive deficits is important to develop for use in preclinical studies. In this context, a new and improved version of the previously described prechiasmatic blood injection mouse model of SAH is presented – referred to as the “double prechiasmatic injection” mouse model of SAH. The model entails two discrete injections (24 hours apart) of determined volumes of heterologous whole blood into the prechiasmatic cistern. This SAH model has a distinct advantage over currently used experimental mouse models of SAH in that it causes highly reproducible injury severity leading to robust neurocognitive deficits 1-month after ictus, along with trackable chronic neuroinflammatory changes. The prechiasmatic double injection model eliminates possible confounding variables by allowing control over the initiation, volume, and rate of hemorrhage with low mortality. It also allows for variation of blood volumes and injection rates to optimize resulting neurocognitive deficits across mouse strains, conditions, and laboratories.

### Prechiasmatic SAH-induced long-term neurobehavioral impairment

**Figure 2:**
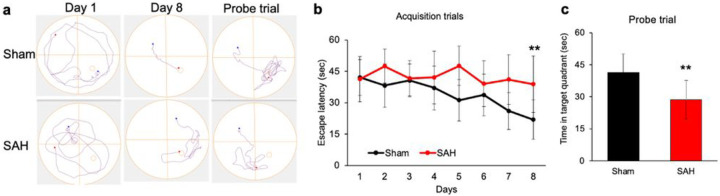
SAH led to impaired spatial learning and memory in the Morris water maze. WT mice underwent SAH or sham. **a**, Representative paths of the two groups of mice. **b**, Escape latency measured as mean Time (s) during training trials. **c**, Time spent by each group in the target quadrant during the probe trial. Data represent the mean ± s.d. The statistical comparison is performed by a two-tailed t-test, **p < 0.01 vs. Sham group; n = 10 per group.

In this mouse model of SAH, a battery of different neurobehavioural paradigms, such as Morris water maze (MWM), Y-maze, novel object recognition (NOR), and fear conditioning (FC) were used for the assessment of long-term neurological functions. In the MWM, independent t-test revealed significant difference (p < 0.01) in the latency to reach the platform on day 8 between sham and SAH mice, indicating the spatial learning and memory impairment in SAH mice (Fig. 2b). Furthermore, the outcome from the probe trial showed a loss in spatial memory retention in SAH mice, compared to sham animals, as concluded by significantly less time spent by SAH mice in target quadrant compared to sham group (Fig. 2c).

**Figure 3:**
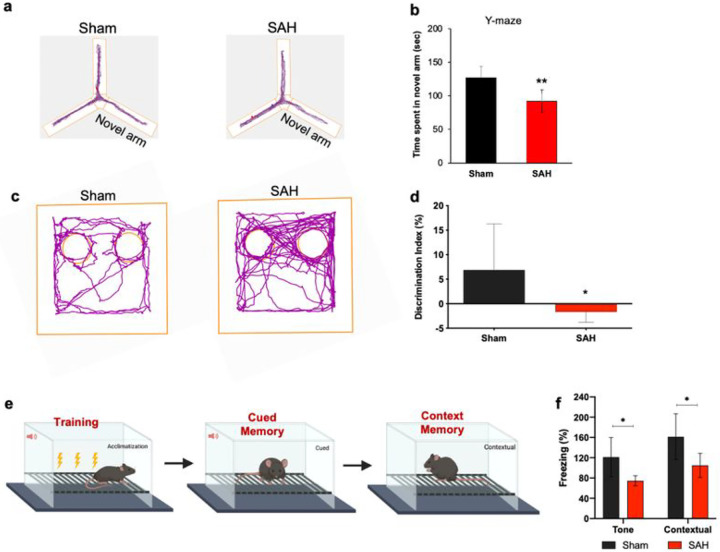
SAH induces behavioral deficits in prechiasmatic double blood injection mouse SAH model. WT mice underwent SAH or sham surgery and were evaluated 30 d post-SAH for cognitive function. **a**, Animal movement traces of sham (Left) and SAH (Right) mice during the test phase. **b**, Novel arm entries in the Y-maze test. **c**, Representative preferential investigation track of sham (Left) SAH (Right) mice for the novel objects. **d**, Differences in the degree of impaired discrimination index following SAH. **e**, Mice were trained with fear conditioning and tested with their freezing responses to tone and context. **f**, Percent freezing of mice during tone and contextual fear conditioning test. Comparison of percentage of freezing of SAH mice with sham controls during tone and contextual fear conditioning tests. Data represent the mean ± s.d. The statistical comparison is performed by a two-tailed t-test, *p < 0.05; **p < 0.01 vs. Sham group; n = 10 per group.

The Y-maze test was performed to test the cognitive function that assesses the natural exploration behavior of rodents. Our results indicated that SAH led to memory impairment in mice compared to sham controls as evidenced by significantly (p < 0.01) less time spent by SAH mice in novel arm compared to sham-operated mice (Fig. 3a,b).

The NOR test was performed to evaluate the perirhinal cortex circuitry, which showed that the brain region is compromised in this mouse model of SAH (Fig. 3c,d). Compared to sham-operated mice, SAH mice showed a significant decline (p < 0.05) in the discriminative index, indicating cognitive impairment after SAH.

Lastly, we performed the FC test, the aversive associative learning and memory assessment of sham and SAH-operated mice. The test concluded impaired tone and contextual conditioning in SAH animals. SAH mice showed significantly reduced percent freezing compared to sham controls in tone test, indicating an injury-induced impairment of amygdala- and hippocampus-associated memory function (Fig. 3e,f).

### Prechiasmatic SAH-induced thromboinflammation

**Figure 4:**
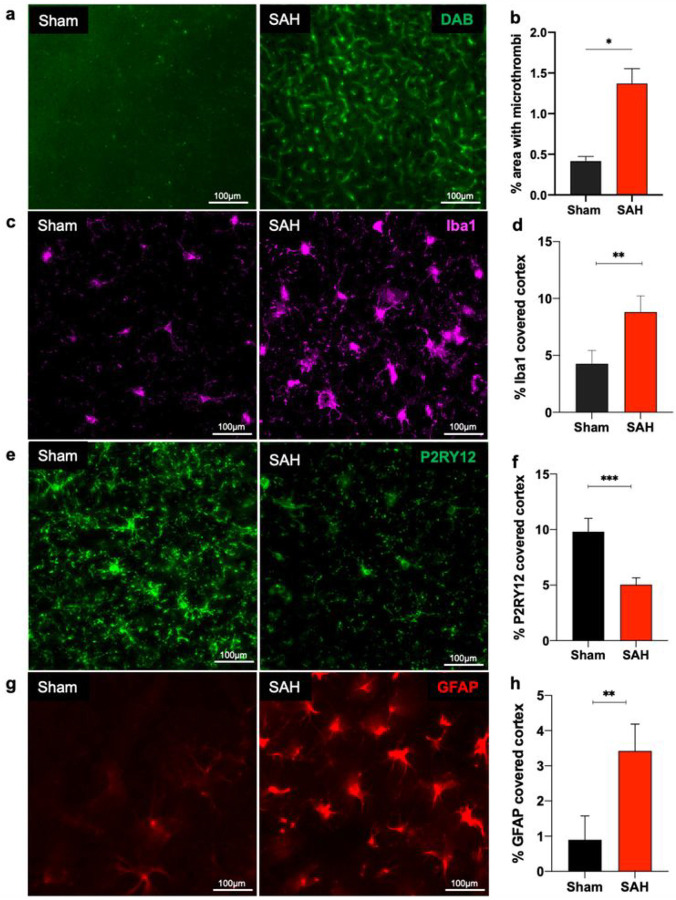
SAH-induced microvascular thrombosis. **a,** Representative images of the fibrinogen-positive microthrombi in mice that underwent sham and SAH surgery. **b,** Brain tissue microthrombosis was determined as percent coverage of ipsilateral parietal cortex. Data represent the mean ± s.d. The statistical comparison is performed by a two-tailed t-test. *p < 0.05 vs. Sham group; n = 5 per group. Each group analyzed four brain sections per animal; scale bar = 100μm.

The SAH-associated microvessel thrombosis and inflammation are described well in the acute and subacute phases but haven’t been reported in the delayed phase following injury to date, nor has it been evaluated for concurrence with microthrombi, neuroinflammation, and delayed neurological deterioration. We are the first to establish this correlation of SAH insult in our prechiasmatic double blood injection mouse model of SAH (Figs. 2,3, and 4). While microvessel thrombi have been observed in endovascular perforation models of SAH due to downstream mechanisms of vessel rupture,[6, 33–35] the blood infusion SAH models offer better insight into the SAH-induced thrombo-inflammation post-ictus. However, no such vessel rupture is present in these experimental models of SAH. The potential cause of microvessel thrombi within the cerebral vasculature following SAH is sustained inflammation. Our prechiasmatic double injection model of SAH showed microthrombi formation (Fig. 4a,b), microgliosis (Fig. 4c,d), and astrocytosis (Fig. 4g,h) in the different regions of the SAH brain at day 30 after injury, indicating the sustained post-ictus influence on histopathological and neurological outcomes.

## Discussion

Long-term neurocognitive deficits are the primary driver of poor quality of life and low rate of return to work in patients who suffer SAH. New treatment strategies to combat the pathophysiological contributors to these long-term cogntivie deficits (including EBI and DCI) are desperately needed, and animal models that more fully recapitulate the pathology and disability seen in SAH patients would greatly aid investigators’ efforts. To date, the lack of a mouse model of SAH that consistently and reproducibly causes trackable long-term neurocogntivie deficits across independent laboratories has been a barrier to identification of novel, effective, and translatable therapies. The rich toolset of genetically modified mice makes this species particularly attractive for such studies.

In the present work, we sought to improve SAH disease modeling by modifying the previously reported pre-chiasmatic injection mouse model. The major modification was to inject heterologous blood into the prechiasmatic cistern in two stages, where the second blood injection was performed 24 h after the first injection. We then conducted a series of neurobehavioral assessments and found that mice with SAH displayed several significant impairments. These impairments included (1) spatial learning and memory impairment in the MWM test, (2) a decline in cognitive ability in the Y-maze test, (3) a compromised recognition memory function in the NOR test, and (4) a significant decrease in memory function in the FC test, as indicated by a decrease in the percentage of freezing to tone and contextual conditioning compared to the sham group.

We also discovered that the neurobehavioral deficits present one month after SAH were strongly associated with chronic inflammation. This association was demonstrated by the percentage area immunostained by Iba-1 (microgliosis) and GFAP (astrocytosis). Additionally, we observed persistent microvascular thrombi after double injection SAH, suggestive of impaired fibrinolytic activity. In total, our findings suggest our prechiasmatic double injection mouse model of SAH could be an important new experimental tool towards identifying and addressing the underlying mechanisms of SAH-induced neurocognitive dysfunction.

Our model has distinct advantages to other available mouse models of SAH. For single blood injection models into the prechiasmatic cistern or cisterna magna, neurocognitive deficits have only been observed up to 2-week after ictus (Table 1) [12, 22, 23], which limits translatability to the human condition. For the endovascular perforation mouse model, disparate results have been reported with one laboratory noting significant neurocognitive deficits one month post-ictus[24] and two other laboratories showing no or minimal neurocognitive deficits one month post-ictus[25] [26]. Important advantages of our mouse model of SAH compared with existing methods are summarized in Table 1.

## Conclusion

The proposed prechiasmatic double injection mouse model offers a promising means for testing the impact of genetically modified strains on the most important endpoint in SAH – long-term neurocognitive deficits. This model offers ease of application, reliability, and high spatiotemporal control of the injected blood volume, and it permits fair comparisons between experimental groups by reducing variability. If replicated by independent laboraties, this mouse model could become the standard for studies examining the pathophysiology of SAH and evaluating potential therapeutic avenues to improve long-term neurobehavioral outcomes. The implementation of such a model would, therefore, provide a valuable translational tool for advancing our understanding of SAH and its treatment.

## Figures and Tables

**Figure 1:**
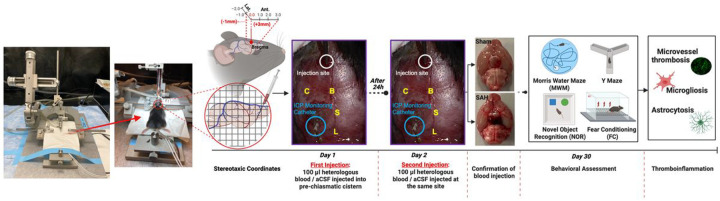
Overview of the protocol for developing and assessing prechiasmatic double blood injection mouse model of subarachnoid hemorrhage (SAH). The protocol includes preparation, pre- and post-procedure management of animals, behavioral assessments at certain time points post-SAH, and finally, euthanasia and injury-induced thromboinflammation analysis.

**Figure 5:** SAH induces sustained neuroinflammation in the delayed phase of the injury. The mouse brain sections were immunostained Iba-1 for microgliosis, P2RY12 for homeostatic microglia, and GFAP for astrocytosis. **a,** Photomicrographs of immunostaining of Iba-1 (a marker for microglia), **c,** P2RY12 (a marker for quiescent microglia) **e,** and GFAP (a marker of astrocyte). **b,d,f,** Quantification of the relative intensity of Iba-1 (**b**), P2RY12 (**d**), and GFAP (**f**). Data represent the mean ± s.d. The statistical comparison is performed by a two-tailed t-test, **p < 0.01; ***p < 0.001 vs. Sham group; n = 4 per group. Each group analyzed four brain sections per animal; scale bar = 100μm.

## Data Availability

The data sets used and/or analyzed during the current study are available from the corresponding author on reasonable request.

## References

[1] Rinkel GJE, Algra A. Long-term outcomes of patients with aneurysmal subarachnoid haemorrhage. Lancet Neurol. 2011. p. 349–56. 21435599 10.1016/S1474-4422(11)70017-5

[2] Macdonald RL, Schweizer TA. Spontaneous subarachnoid haemorrhage. Lancet. 2017. p. 655–66. 27637674 10.1016/S0140-6736(16)30668-7

[3] Diringer MN. Management of aneurysmal subarachnoid hemorrhage. Crit Care Med [Internet]. 2009;37:432–40. https://www.scopus.com/inward/record.uri?eid=2-s2.0-67650392042&doi=10.1097%2FCCM.0b013e318195865a&partnerID=40&md5=0448d00dc43579cfc17ce936dfe05265 19114880 10.1097/CCM.0b013e318195865a PMC2820121

[4] Titova E, Ostrowski RP, Zhang JH, Tang J. Experimental models of subarachnoid hemorrhage for studies of cerebral vasospasm. Neurol Res. 2009. 10.1179/174313209X382412 19108759

[5] Parra A, McGirt MJ, Sheng H, Laskowitz DT, Pearlstein RD, Warner DS. Mouse model of subarachnoid hemorrhage associated cerebral vasospasm: Methodological analysis. Neurol Res. 2002. 10.1179/016164102101200276 12117325

[6] Diwan D, Vellimana AK, Aum DJ, Clarke J, Nelson JW, Lawrence M Sirtuin 1 Mediates Protection Against Delayed Cerebral Ischemia in Subarachnoid Hemorrhage in Response to Hypoxic Postconditioning. J Am Heart Assoc [Internet]. 2021;10:e021113. https://www.ahajournals.org/doi/10.1161/JAHA.121.021113. 34622677 10.1161/JAHA.121.021113 PMC8751859

[7] Vellimana AK, Aum DJ, Diwan D, Clarke JV, Nelson JW, Lawrence M SIRT1 mediates hypoxic preconditioning induced attenuation of neurovascular dysfunction following subarachnoid hemorrhage. Exp Neurol [Internet]. 2020;334:113484. https://linkinghub.elsevier.com/retrieve/pii/S0014488620303150. 33010255 10.1016/j.expneurol.2020.113484 PMC8908895

[8] Milner E, Johnson AW, Nelson JW, Harries MD, Gidday JM, Han BH HIF-1a Mediates Isoflurane-Induced Vascular Protection in Subarachnoid Hemorrhage. Ann Clin Transl Neurol. 2015;2. 10.1002/acn3.170 PMC4402079 25909079

[9] Vellimana AK, Zhou ML, Singh I, Aum DJ, Nelson JW, Harris GR, Minocycline protects against delayed cerebral ischemia after subarachnoid hemorrhage via matrix metalloproteinase-9 inhibition. Ann Clin Transl Neurol. 2017;4:865–76. 29296615 10.1002/acn3.492 PMC5740245

[10] Marbacher S, Fandino J, Kitchen ND. Standard intracranial in vivo animal models of delayed cerebral vasospasm. Br J Neurosurg. 2010;24. 10.3109/02688691003746274 20726750

[11] Chung DY, Oka F, Jin G, Harriott A, Kura S, Aykan SA, Subarachnoid hemorrhage leads to early and persistent functional connectivity and behavioral changes in mice. J Cereb Blood Flow Metab. 2021;41:975–85. 32936728 10.1177/0271678X20940152 PMC8054726

[12] Pedard M, El Amki M, Lefevre-Scelles A, Compère V, Castel H. Double Direct Injection of Blood into the Cisterna Magna as a Model of Subarachnoid Hemorrhage. Journal of Visualized Experiments [Internet]. 2020;2020. https://www.jove.com/t/61322/double-direct-injection-of-blood-into-the-cisterna-magna-as-a-model-of-subarachnoidhemorrhage. 10.3791/61322 32925881

[13] Prunell GF, Mathiesen T, Svendgaard NA. A new experimental model in rats for study of the pathophysiology of subarachnoid hemorrhage. NeuroReport. 2002;13. 10.1097/00001756-200212200-00034 12499866

[14] Raslan F, Albert-Weißenberger C, Westermaier T, Saker S, Kleinschnitz C, Lee JY. A modified double injection model of cisterna magna for the study of delayed cerebral vasospasm following subarachnoid hemorrhage in rats. Exp Transl Stroke Med. 2012;4. 10.1186/2040-7378-4-23 PMC3552945 23194464

[15] Leclerc JL, Garcia JM, Diller MA, Carpenter A-M, Kamat PK, Hoh BL A Comparison of Pathophysiology in Humans and Rodent Models of Subarachnoid Hemorrhage. Front Mol Neurosci [Internet]. 2018;11. https://www.scopus.com/inward/record.uri?eid=2-s2.0-85046907469&doi=10.3389%2Ffnmol.2018.00071&partnerID=40&md5=99cbdfa1fdba9a5a9345566f8ce743f0 10.3389/fnmol.2018.00071 PMC5875105 29623028

[16] Sehba FA, Pluta RM. Aneurysmal Subarachnoid Hemorrhage Models: Do They Need a Fix? Stroke Res Treat [Internet]. 2013;2013:1–13. http://www.hindawi.com/journals/srt/2013/615154/. 10.1155/2013/615154 PMC3710594 23878760

[17] Attia MS, Macdonald R. Anterior circulation model of subarachnoid hemorrhage in mice [Internet]. Acta Neurochir Suppl (Wien). 2015. pp. 311–4. https://www.scopus.com/inward/record.uri?eid=2-s2.0-84921790111&doi=10.1007%2F978-3-319-04981-6_53&partnerID=40&md5=c21d6aeb993b8360609823026f9ecae3 10.1007/978-3-319-04981-6_53 25366643

[18] Strbian D, Durukan A, Tatlisumak T. Rodent Models of Hemorrhagic Stroke. Curr Pharm Des. 2008;14. 10.2174/138161208783497723 18289061

[19] Hu Q, Vakhmjanin A, Li B, Tang J, Zhang JH. Hyperbaric oxygen therapy fails to reduce hydrocephalus formation following subarachnoid hemorrhage in rats. Med Gas Res. 2014;4. 10.1186/2045-9912-4-12 PMC4134116 25132956

[20] Sasaki T, Hoffmann U, Kobayashi M, Sheng H, Ennaceur A, Lombard FW, Long-Term Cognitive Deficits After Subarachnoid Hemorrhage in Rats. Neurocrit Care. 2016;25:293–305. 26896093 10.1007/s12028-016-0250-1

[21] Boyko M, Azab AN, Kuts R, Gruenbaum BF, Gruenbaum SE, Melamed I The neuro-behavioral profile in rats after subarachnoid hemorrhage. Brain Res [Internet]. 2013;1491:109–16. https://linkinghub.elsevier.com/retrieve/pii/S0006899312017519. 23123210 10.1016/j.brainres.2012.10.061

[22] Boettinger S, Kolk F, Broessner G, Helbok R, Pfausler B, Schmutzhard E Behavioral characterization of the anterior injection model of subarachnoid hemorrhage. Behavioural brain research [Internet]. 2017;323:154–61. https://linkinghub.elsevier.com/retrieve/pii/S0166432817302206. 28174030 10.1016/j.bbr.2017.02.004

[23] Turan N, Heider RA, Nadeem M, Miller BA, Wali B, Yousuf S Neurocognitive Outcomes in a Cisternal Blood Injection Murine Model of Subarachnoid Hemorrhage. J Stroke Cerebrovasc Dis. 2020;29. 10.1016/j.jstrokecerebrovasdis.2020.105249 33066928

[24] Regnier-Golanov AS, Gulinello M, Hernandez MS, Golanov EV, Britz GW. Subarachnoid Hemorrhage Induces Sub-acute and Early Chronic Impairment in Learning and Memory in Mice. Transl Stroke Res. 2022;13. 10.1007/s12975-022-00987-9 35260988

[25] Milner E, Holtzman JC, Friess S, Hartman RE, Brody DL, Han BH Endovascular Perforation Subarachnoid Hemorrhage Fails to Cause Morris Water Maze Deficits in the Mouse. Journal of Cerebral Blood Flow & Metabolism [Internet]. 2014;34:e1–9. http://journals.sagepub.com/doi/10.1038/jcbfm.2014.108. 10.1038/jcbfm.2014.108 PMC4158664 24938403

[26] Fanizzi C, Sauerbeck AD, Gangolli M, Zipfel GJ, Brody DL, Kummer TT. Minimal long-term neurobehavioral impairments after endovascular perforation subarachnoid hemorrhage in mice. Sci Rep [Internet]. 2017;7:7569. http://www.nature.com/articles/s41598-017-07701-y. 28790425 10.1038/s41598-017-07701-y PMC5548778

[27] Mehla J, Lacoursiere SG, Lapointe V, McNaughton BL, Sutherland RJ, McDonald RJ Age-dependent behavioral and biochemical characterization of single APP knock-in mouse (APPNL-G-F/NL-G-F) model of Alzheimer’s disease. Neurobiol Aging. 2019;75. 10.1016/j.neurobiolaging.2018.10.026 30508733

[28] Mehla J, Singh I, Diwan D, Nelson JW, Lawrence M, Lee E STAT3 inhibitor mitigates cerebral amyloid angiopathy and parenchymal amyloid plaques while improving cognitive functions and brain networks. Acta Neuropathol Commun. 2021;9. 10.1186/s40478-021-01293-5 PMC8672532 34911575

[29] Mehla J, Deibel SH, Karem H, Hossain S, Lacoursiere SG, Sutherland RJ Dramatic impacts on brain pathology, anxiety, and cognitive function in the knock-in APPNL-G-F mouse model of Alzheimer disease following long-term voluntary exercise. Alzheimers Res Ther. 2022;14. 10.1186/s13195-022-01085-6 PMC9526288 36180883

[30] Jafari Z, Mehla J, Kolb BE, Mohajerani MH. Prenatal noise stress impairs HPA axis and cognitive performance in mice. Sci Rep. 2017;7. 10.1038/s41598-017-09799-6 PMC5585382 28874680

[31] Mehla J, Lacoursiere S, Stuart E, McDonald RJ, Mohajerani MH. Gradual Cerebral Hypoperfusion Impairs Fear Conditioning and Object Recognition Learning and Memory in Mice: Potential Roles of Neurodegeneration and Cholinergic Dysfunction. J Alzheimer’s Disease. 2018;61. 10.3233/JAD-170635 29154281

[32] Aum DJ, Vellimana AK, Singh I, Milner E, Nelson JW, Han BH A novel fluorescent imaging technique for assessment of cerebral vasospasm after experimental subarachnoid hemorrhage. Sci Rep [Internet]. 2017;7:9126. http://www.nature.com/articles/s41598-017-09070-y. 28831103 10.1038/s41598-017-09070-y PMC5567362

[33] Sabri M, Ai J, Lakovic K, D’abbondanza J, Ilodigwe D, MacDonald RL. Mechanisms of microthrombi formation after experimental subarachnoid hemorrhage. Neuroscience [Internet]. 2012;224:26–37. https://linkinghub.elsevier.com/retrieve/pii/S0306452212008172. 22902542 10.1016/j.neuroscience.2012.08.002

[34] Dienel A, Kumar TP, Blackburn SL, McBride DW. Role of platelets in the pathogenesis of delayed injury after subarachnoid hemorrhage. J Cereb Blood Flow Metab. 2021. 10.1177/0271678X211020865 PMC8756481 34112003

[35] McBride DW, Blackburn SL, Peeyush Kumar T, Matsumura K, Zhang JH. The role of thromboinflammation in delayed cerebral ischemia after subarachnoid hemorrhage. Front Neurol. 2017. 10.3389/fneur.2017.00555 PMC5660311 29109695

